# Supplementary figures and images for: Regulation of soldier caste differentiation by microRNAs in Formosan subterranean termite (Coptotermes formosanus Shiraki)
Source: PeerJ. 2024 Feb 29;12:e16843. doi: 10.7717/peerj.16843 (PMC10909360; doi:10.7717/peerj.16843)

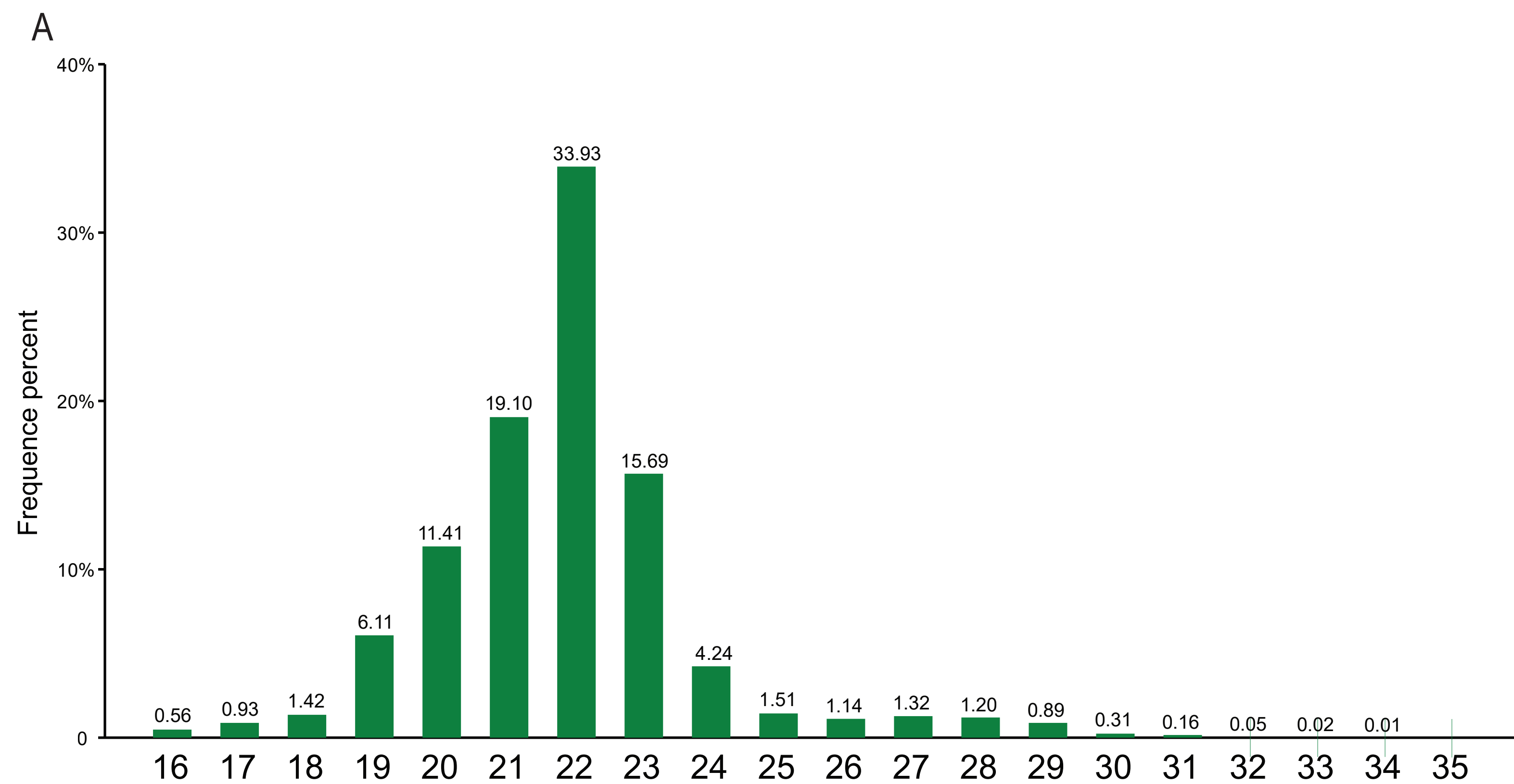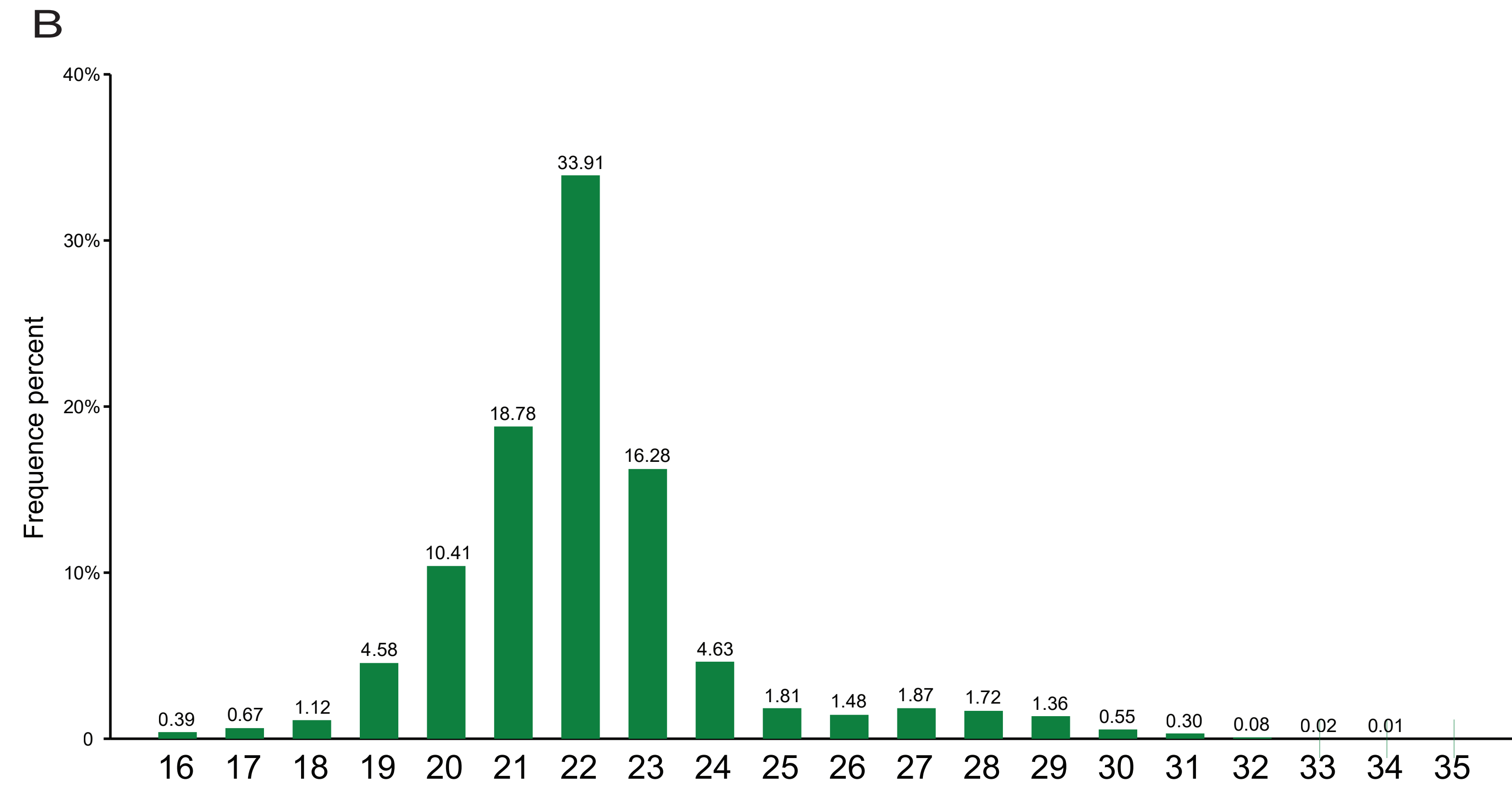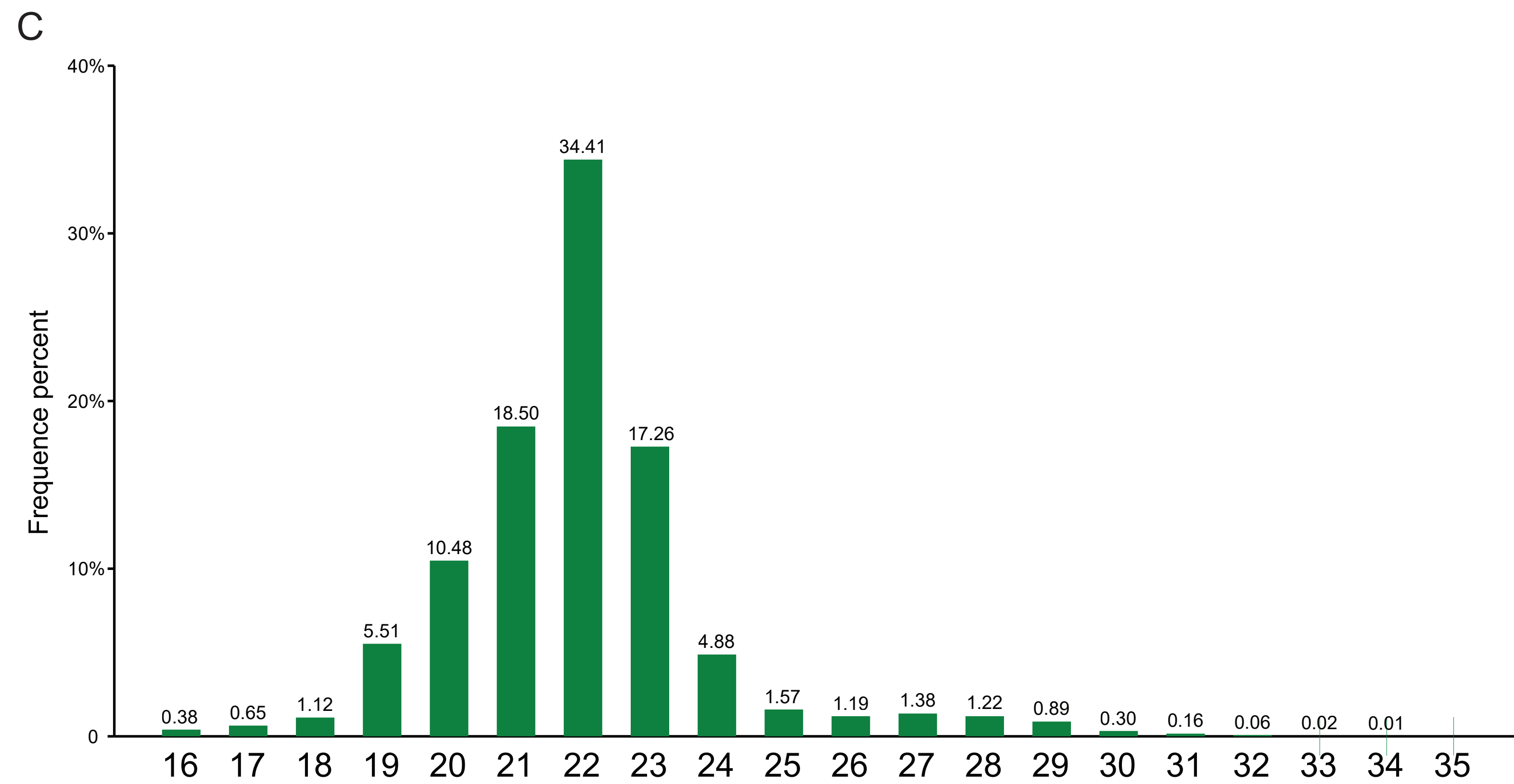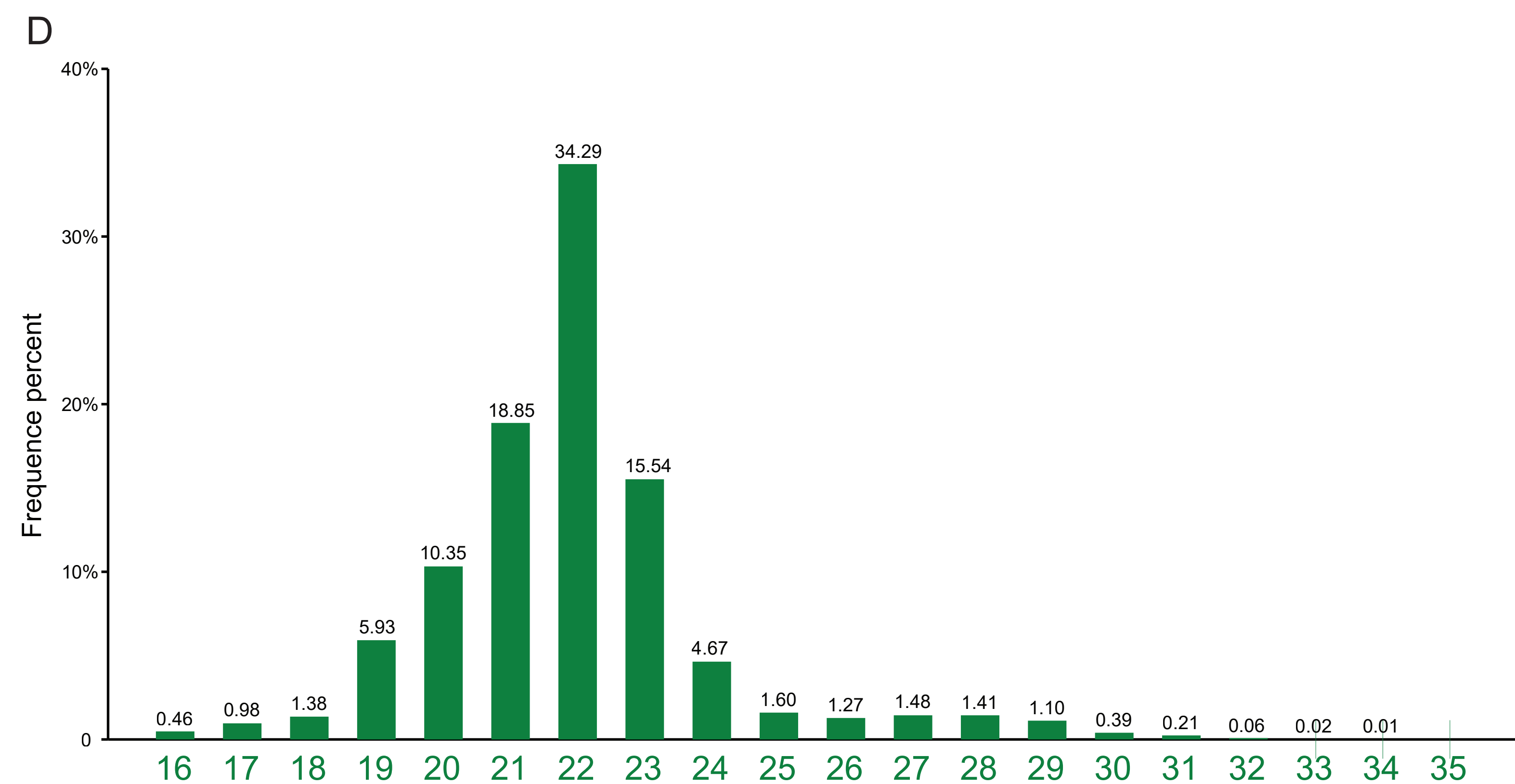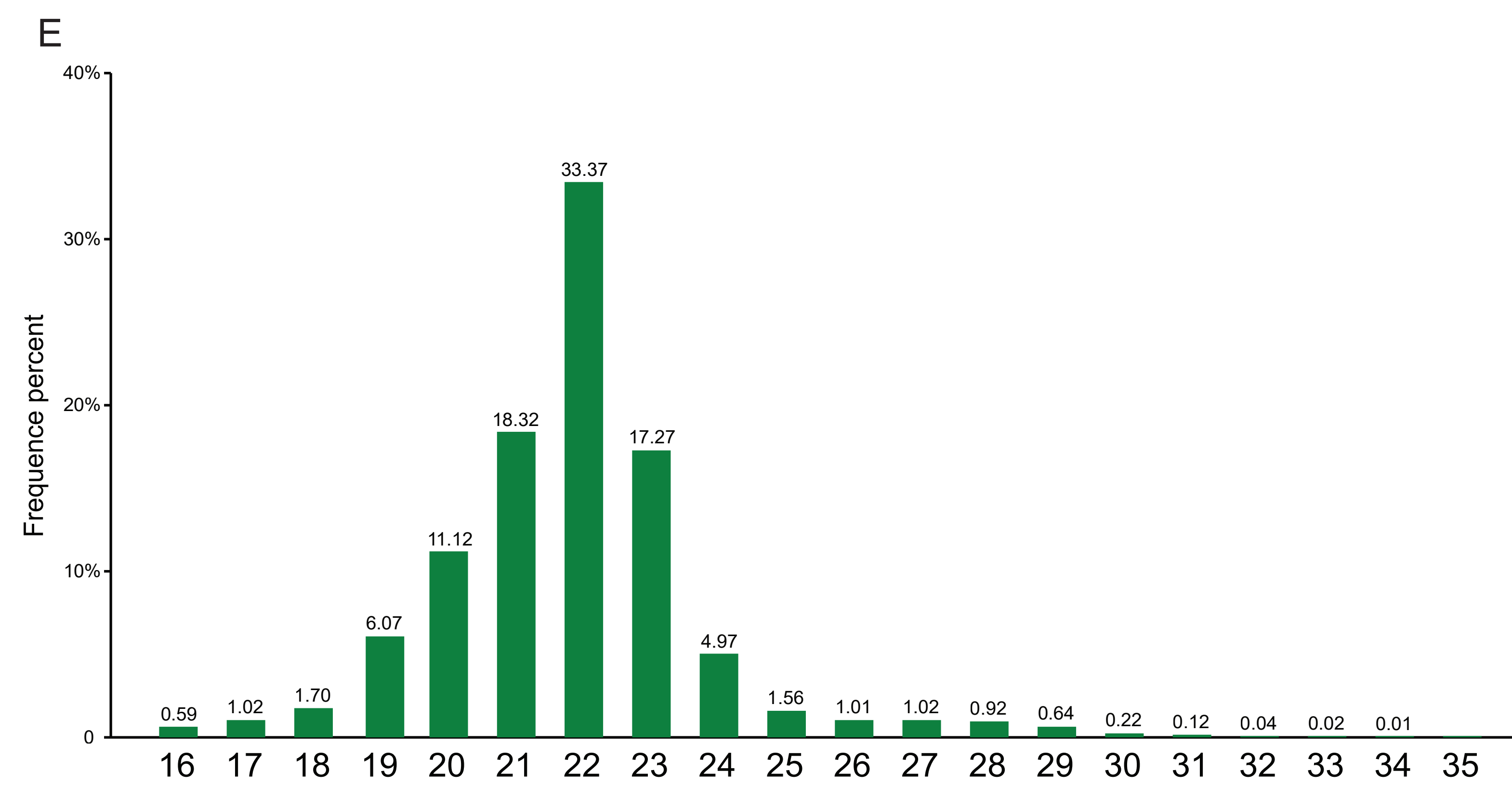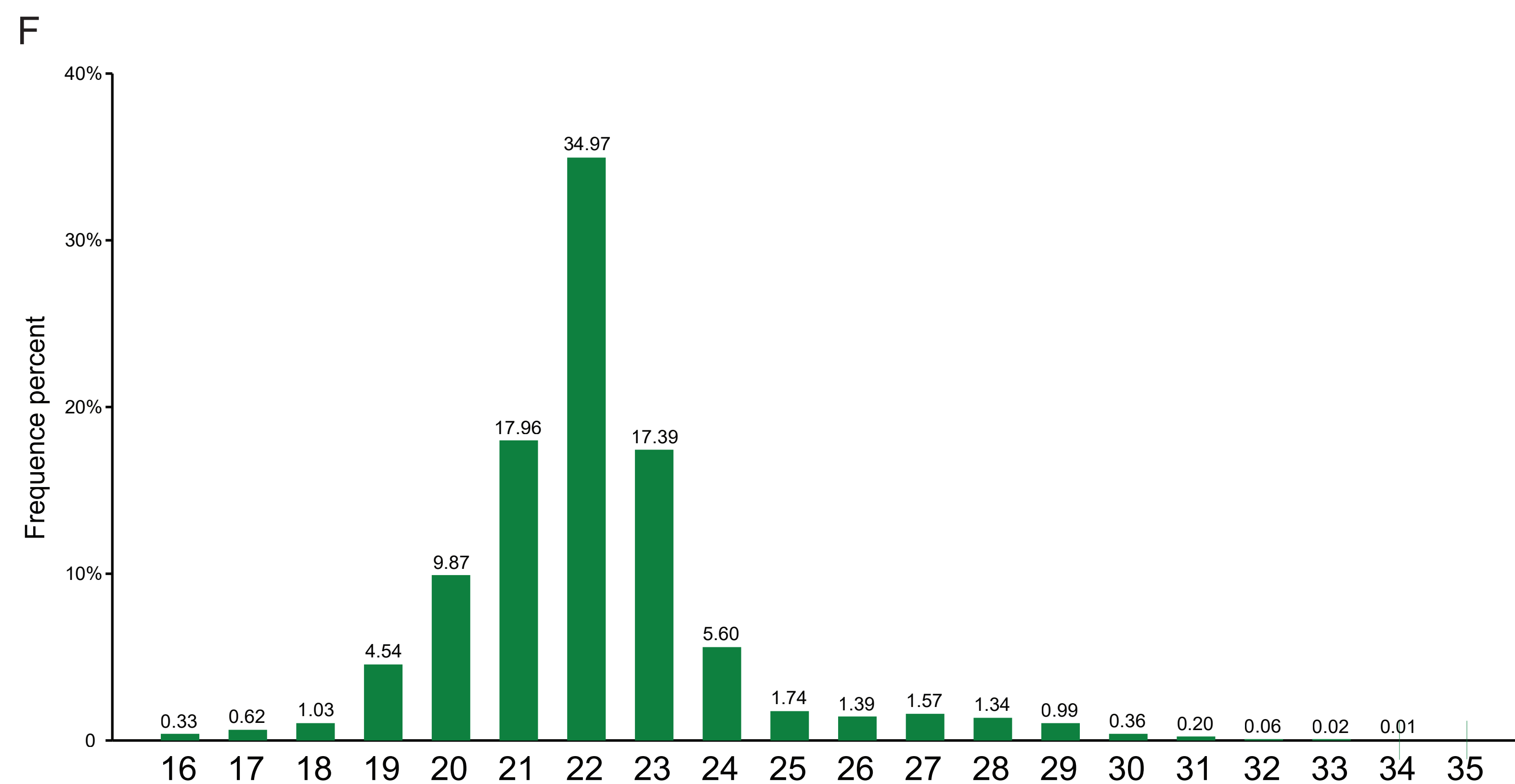

Supplement: Supplemental Information 2 — (A)–(F) are the length distribution of the tags from C-1, C-2, C-3, M-1, M-2, M-3, respectively. C1–C3 are the three replicates from the control group (C) and M1–M3 are the three replicates from the methoprene-treated group (M). [file peerj-12-16843-s002.pdf]
